# Brazilian Protocol for Sexually Transmitted infections, 2020: pelvic inflammatory disease

**DOI:** 10.1590/0037-8682-602-2020

**Published:** 2021-05-17

**Authors:** Maria Luiza Bezerra Menezes, Paulo Cesar Giraldo, Iara Moreno Linhares, Neide Aparecida Tosato Boldrini, Mayra Gonçalves Aragon

**Affiliations:** 1 Universidade de Pernambuco, Departamento Materno-Infantil, Recife, PE, Brasil.; 2 Universidade Estadual de Campinas, Departamento de Tocoginecologia, Campinas, SP, Brasil.; 3 Universidade de São Paulo, Disciplina de Ginecologia, Departamento de Obstetrícia e Ginecologia, São Paulo, SP, Brasil.; 4 Universidade Federal do Espírito Santo, Departamento de Ginecologia e Obstetrícia, Vitória, ES, Brasil.; 5 Ministério da Saúde, Secretaria de Vigilância em Saúde, Brasilia, DF, Brasil.; 6 Universidade Federal do Espírito Santo, Programa de Pós-Graduação em Doenças Infecciosas, Vitória, ES, Brasil.

**Keywords:** Pelvic infection, Pelvic pain, Ectopic pregnancy, Infertility, Chlamydia trachomatis, Neisseria gonorrhoeae

## Abstract

Pelvic Inflammatory Disease is a topic included in the Clinical Protocol and Therapeutic Guidelines for Comprehensive Care for People with Sexually Transmitted Infections, published by the Brazilian Ministry of Health in 2020. Pelvic inflammatory disease is an upper female genital tract acute infection due to canalicular spread of endogenous cervicovaginal microorganisms and especially the sexually transmitted microorganisms. Standing out among the etiological agents involved are *Chlamydia trachomatis* and *Neisseria gonorrhoeae*. The main sequelae are chronic pelvic pain, infertility, and ectopic pregnancy. Clinical diagnosis is the most important practical approach. Antibiotic treatment must start immediately after the clinical suspicion. Guidelines for health service managers and health professionals on diagnostic tests, treatment, follow-up, counseling, notification, handling sexual partners and special populations are described. Given the increased availability of the molecular biology techniques in Brazil, *C. trachomatis* and *N. gonorrhoeae* screening are recommended as a disease prevention strategy.

## Highlighted excerpt

Pelvic inflammatory disease is one of the most significant sexually transmitted infections, and in most cases, it is a main consequence of cervicitis.

## FOREWORD

This article addresses pelvic inflammatory disease, included in the Clinical Protocol and Therapeutic Guidelines (PCDT) for Comprehensive Care for People with Sexually Transmitted Infections (STI), published by the Health Surveillance Secretariat of the Brazilian Ministry of Health. For the development of the PDCT, selection and analysis of available evidence were performed, followed by discussions with specialists. The PDCT was approved by the National Committee for Technology Incorporation to the Brazilian National Health System (Conitec)^1^ and updated by the panel of specialists in STI in 2020.

## EPIDEMIOLOGY

Pelvic inflammatory disease is defined as the clinical inflammatory and infectious syndrome arising from the ascent of microorganisms from the lower genital tract (vagina and cervix) to the upper genital tract, which may harm the endometrium, tubes, ovaries, pelvic peritoneum, and adjacent structures. Consequently, endometritis, salpingitis, oophoritis, and pelvic peritonitis can arise, depending on the infection's extent. Dissemination occurs predominantly through the canalicular route^2^ ^,^ ^3^.

Although the classical pelvic inflammatory disease definition includes only canalicular and spontaneous microorganism dissemination, not associated with surgical or procedures or pregnancy^3^, intrauterine devices (IUD) insertion, endometrium biopsy, curettage, among others, are also currently considered as responsible for the syndrome^2^.

Pelvic inflammatory disease is one of the most significant sexually transmitted infections, and a principal negative consequence of cervicitis. It is estimated that there is one case of pelvic inflammatory disease for every eight to ten women with *Chlamydia trachomatis* cervicitis^3^. The absence of rapid diagnosis and treatment or inadequate treatment increases the risk of severe complications, with negative consequences for women's health, as well as economic and social costs. Most sequelae involve infertility, ectopic pregnancy, and chronic pelvic pain^4^ ^-^ ^6^. It has been reported that, after seven years from the first episode, 21.3% of women presented recurrence, 19% developed infertility, and 42.7% experienced chronic pelvic pain^7^.

Existing prevalence data are underestimated, since pelvic inflammatory disease is not compulsorily notified, and, for this reason, the number of affected women is unknown^8^. Also, many cases present mild or few clinical symptoms which are not noticed. Studies indicate that 10% to 40% of women with cervicitis caused by *N. gonorrhoeae* or *C. trachomatis* develop the pelvic inflammatory disease^9^ ^,^ ^10^. In Brazil, the true prevalence of the disease is unknown. Using Brazilian National Health System Hospital Information System, the hospitalization number of women with pelvic inflammatory disease from January 2005 to August 2006 was obtained. The hospitalization average per year was 45,343 cases. However, it is essential to highlight that such data reflects only severe cases of the syndrome, those that required hospital care, and they represent only a small fraction of the affected women since in most of them the infection elicits only light or moderate symptoms or is asymptomatic^11^.

In a study conducted in the United States, 4.4% of 1,171 sexually active women between 18 and 44 years old had symptoms suggesting pelvic inflammatory disease^12^. From this result, it was estimated that for the period 2013-2014, 2.5 million women had pelvic inflammatory disease in the United States^13^. Another study suggests that 800,000 cases of the disease occur per year in that country^14^. Sutton et al. (2005) estimated 1.2 million medical visits yearly due to pelvic inflammatory disease in developed countries^15^.

The endocervix is classically considered a protecting barrier in the upper genital tract. Endocervical infection with sexually transmitted pathogens breaks this barrier providing access of vaginal bacteria to the upper genital organs, infecting endometrium, endosalpinx, pelvic peritoneum, and subjacent stroma^2^ ^,^ ^3^ ^,^ ^16^. The reasons for which bacteria in the lower genital tract cause pelvic inflammatory disease in only some women are not entirely known. Still, they may be associated with genetic variations, retrograde menstruation, immune response, bacterial load and menstrual cycle hormonal oscillations, considering that the menstrual cervix mucous is less bacteriostatic^17^ ^-^ ^19^. The infection's progression by anaerobic agents determines a higher oxygen consumption and a local oxyreduction potential decrease, which, alongside tissue devitalization, provides an environment of microaerophilia or even anaerobiosis (Monif theory)^16^. In such environment, the microorganisms that reached the upper genital tract start a slow-growing phase, and opportunistic anaerobic agents develop. The result is a polymicrobial infectious condition^2^ ^,^ ^3^ ^,^ ^16^.

From the observation that two-thirds of women with STI did not have any previous history of treatment for pelvic inflammatory disease, the concept of subclinical pelvic inflammatory disease was proposed, being as common as the clinical disease, and presenting the same etiology^6^ ^,^ ^20^.

**FIGURE 1:**
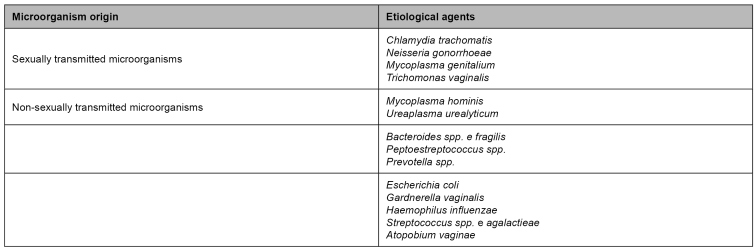
Etiological agents of pelvic inflammatory disease.

**Source:** adapted from Clinical Protocol and Treatment Guidelines for Comprehensive Care for People with Sexually Transmitted Infections, 2020^34^.

Most cases arise from sexually transmitted pathogenic agents, such as *N. gonorrhoeae* and *C. trachomatis* ^7^ ^,^ ^21^ ^,^ ^22^. A minor fraction of acute disease is not sexually transmitted but associated with germs that colonize the lower genital tract or enteric ones such as *Mycoplasma hominis, Ureaplasma urealyticum, Peptococcus spp., Peptoestreptococcus spp., Bacteroides spp., Escherichia coli, Streptococcus agalactiae,* and *Campylobacter spp*., in addition to respiratory pathogens (for example, *Haemophilus influenzae, Streptococcus pneumoniae,* Group A *streptococci* and *Staphylococcus aureus*)^23^ ^-^ ^27^. Facultative aerobic organisms in the microbiota are deemed potentially causing agents^27^. Etiological agents of pelvic inflammatory disease are listed in Figure 1.

The use of culture methods for microorganisms identification made it possible to understand better the composition of the healthy vaginal microbiota, in mostly women comprised of one or more species of *Lactobacillus*, which act to protect the vaginal environment^28^. Unbalanced status, such as bacterial vaginosis, which comprises a reduction or lack of *Lactobacillus sp*., and increase in concentrations and variability of anaerobic microorganisms such as *Gardnerella vaginalis* and *Mycoplasma sp*., can contribute to the migration of microorganisms to the upper genital tract. It was already shown that bacterial vaginosis doubles the risk of pelvic inflammatory disease^18^ ^,^ ^29^ ^,^ ^30^.

The microorganism insulation in the upper genital tract has been associated with disease stages. However, studies using molecular methods have shown microorganisms in healthy women's endometrium, including *Lactobacillus* species*, Mycoplasma hominins, G. vaginalis, and Enterobacter sp*., among others^31^ ^,^ ^32^. However, although healthy women can host such microorganisms, their role in health and disease stages is still unknown. The interactions between the infectious agent and genital tract immunity are probably determinant for the permanence in the health or changes to a disease stage. In addition, molecular techniques have identified new microorganisms, such as *Atopobium vaginae*. Studies have also identified new bacteria species and genera in the upper genital tract of women with pelvic inflammatory disease. Therefore, questions about the presence of a healthy microbiota in the upper genital tract and the possibility of mechanisms predisposing to physiologic vaginal flora unbalance that also predispose to infection need to be clarified. Such answers will undoubtedly lead to investigations on the best approach for diagnosis and treatment of pelvic inflammatory disease, as well as most efficient ways to prevent it^33^.

Risk factors for pelvic inflammatory disease include:^34^ 1) age group, as adolescents with multiple sexual partners, due to biological and behavioral factors characteristic in this phase, present risks three times greater for the development of acute pelvic inflammatory disease in comparison with women older than 25 years of age^35^ ^,^ ^36^, regardless of education level and family income; 2) sexual behavior with multiple partners, early start of sexual activities and new partners^35^; 3) using IUD, as women using such device present a slightly higher risk of pelvic inflammatory disease in the first 20 days of insertion, regardless of the type of IUD inserted - copper or levonorgestrel release^37^. This risk is reduced in women treated for STI before IUD insertion^38^.

## CLINICAL ASPECTS

Clinical diagnosis is still the principal approach to pelvic inflammatory disease, despite the wide specter of clinical presentations. Around 65% of the cases can be oligosymptomatic and asymptomatic and later present infertility caused by tubal factors^39^. The time course is usually acute, developing for several days. However, a more extensive manifestation can take place, from weeks to months. The typical disease symptoms are fever, abdominal pain, pelvic pain, dyspareunia, vaginal discharge, and dysuria or frequent urination^39^ ^,^ ^40^. Abnormal uterine bleeding (post-intercourse bleeding, intermenstrual bleeding, and menorrhagia) in one-third or more of the cases is observed. Recent abdominal or pelvic pain, or both, intensifying during intercourse or vigorous movement, can be the only symptom. Pain starting during or right after menstruation is particularly suggestive^41^. Only a minority develop peritonitis or pelvic abscess, which generally manifest through intense pain, higher sensitivity to the examination, and systemic characteristics, such as fever^41^ ^-^ ^43^. More rarely, an extension up to the liver capsule, causing perihepatitis (Fitz-Hugh Curtis syndrome) or sepsis, or both can occur^27^ ^,^ ^44^.

The clinical examination must include vital sign assessment; abdominal examination; vaginal speculum examination, with cervical inspection for friability (easy bleeding) and cervical mucopurulent discharge; bimanual vaginal examination, with cervical mobilization; and adnexal palpation (ovaries and uterine tube)^42^.

The sequelae are chronic pelvic pain, ectopic pregnancy, and infertility. Around 25% of the women with pelvic inflammatory disease will present chronic pelvic pain, 10% to 50% will be infertile, and 15% to 60% will have an ectopic pelvic inflammatory illness, generally caused by scars and adherence in the fallopian tubes^7^ ^,^ ^45^ ^,^ ^46^. Such proportions increase typically with the number of infections, being very high in parts of Africa, Asia, and South America, where healthcare services are not easily accessible^47^ ^,^ ^48^. There are also reports on pelvic inflammatory disease associated with a higher risk of cerebrovascular accident, ovary cancer, and acute small bowel obstruction^47^ ^-^ ^50^.

## DIAGNOSIS

**FIGURE 2:**
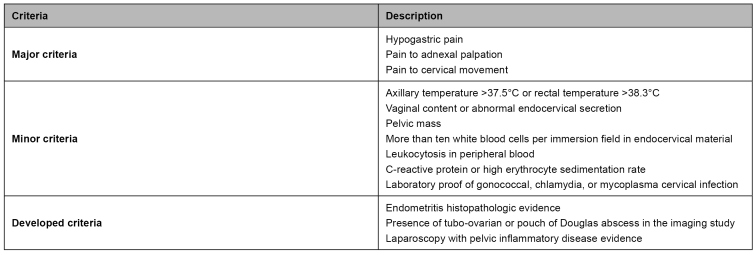
Pelvic inflammatory disease diagnosis criteria.

**Source:** adapted from Clinical Protocol and Treatment Guidelines for Comprehensive Care for People with Sexually Transmitted Infections, 2020^34^.

Clinical diagnosis for pelvic inflammatory disease suspicion is conducted upon three major criteria associated with one minor criterion or one developed criterion^34^, presented in Figure 2.

Laboratory and image examinations help in diagnosis and severity assessment and are central in oligosymptomatic cases. However, in case of high clinical suspicion, treatment must not be delayed. 

The following laboratory tests are recommended: complete blood count; erythrocyte sedimentation rate; C-reactive protein; bacterioscopic examination for bacterial vaginosis; endocervical swab culture with antibiogram; molecular biology for *N. gonorrhoeae* and *C. trachomatis* in material from the endocervix, urethra, laparoscopy, or culdocentesis; qualitative urine examination and urine culture, for dismissing urinary tract infection; hemoculture; pregnancy test, for rejecting the diagnosis of ectopic pregnancy; and imaging exams^51^. Serological exams for *C. trachomatis* are not recommended for pelvic inflammatory disease diagnosis.

Pelvic ultrasound is the preferential imaging exam, as it is accessible and noninvasive, mainly for evaluating possible associated complications, such as tubo-ovarian abscesses, and for excluding differential diagnosis^52^. In pelvic inflammatory disease, the main suspicious sonographic finding is a thin layer of liquid filling the tubes, with or without free fluid in the pelvis. Tomography and resonance examinations can help in differential diagnosis of peritonitis^42^ ^,^ ^51^.

Laparoscopy represents an accurate salpingitis diagnosis resource, enabling a complete bacteriologic diagnosis. However, it does not detect endometritis and less intense tube inflammation, and, thus, its daily use is not justifiable in the disease's initial phase, considering low sensitivity^18^ ^,^ ^19^ and associated morbidity^41^ ^-^ ^43^. Laparoscopy had its great merit in the 1980s, as it led to the Gainesville clinical classification^53^; in practice, it still very used according to clinical and ultrasound findings, being necessary for treatment guidance, to wit: stage I - salpingitis without peritonitis; stage II - salpingitis with peritonitis; stage III - the presence of tubo-ovarian complex, divided in A (hydrosalpinx) and B (tubo-ovarian abscess); stage IV - ruptured tubo-ovarian abscess; and stage V - any of above, associated to genital tuberculosis.

In differential diagnosis, the list includes ectopic pregnancy, acute appendicitis, diverticulitis, urinary tract infection, ureteral lithiasis, ovarian cyst or uterine myoma torsion, ovarian cyst rupture, endometriosis, and ruptured endometrioma, among others^2^ ^,^ ^16^ ^,^ ^22^.

## TREATMENT

**FIGURE 3:**
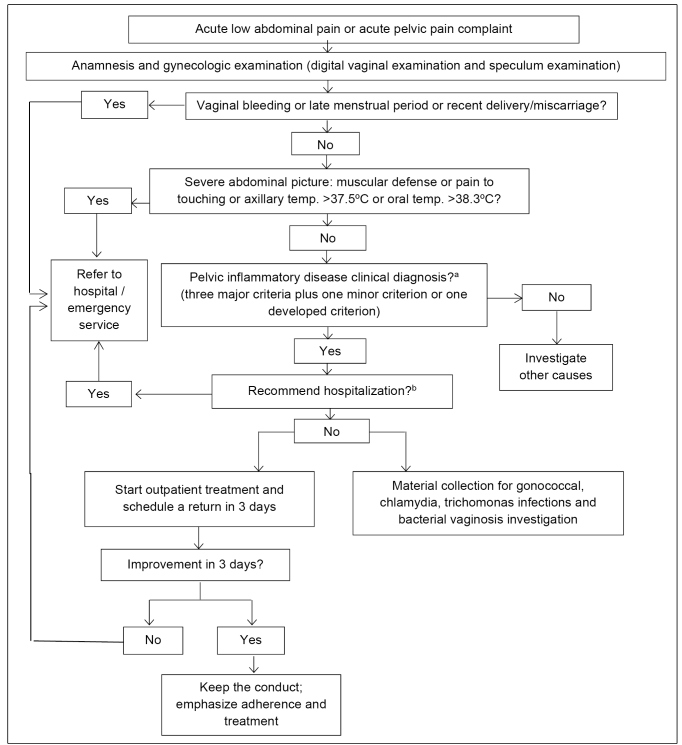
Flowchart for pelvic inflammatory disease clinical management.

**Source:** adapted from Clinical Protocol and Treatment Guidelines for Comprehensive Care for People with Sexually Transmitted Infections,2020^34^.

**Notes:** a) Refer to Figure 2 - Pelvic inflammatory disease diagnosis criteria; b) Refer to Figure 4 - Criteria for recommending pelvic inflammatory disease hospital treatment.

Figure 3 presents guidance for pelvic inflammatory disease outpatient clinical management and indicates the need for urgency evaluation or hospital treatment^34^.

Treatment must start immediately, aiming at avoiding late complications, such as infertility, ectopic pregnancy, and chronic pelvic pain^54^. Treatment of other common pelvic pain causes (ectopic pregnancy, acute appendicitis, ovarian cyst, and functional pain) is unlikely to be harmed by antimicrobial therapy for pelvic inflammatory disease^42^. In addition to antibiotics, analgesic and anti-inflammatory drugs can be used for decreasing symptomatology.

**FIGURE 4:**
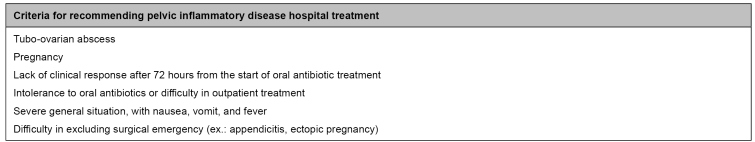
Criteria for recommending pelvic inflammatory disease hospital treatment.

**Source:** adapted from Clinical Protocol and Treatment Guidelines for Comprehensive Care for People with Sexually Transmitted Infections,2020^34^.

Outpatient treatment applies to women that present light clinical pictures without signs of pelvic peritonitis (Gainesville Stage I)^53^. Other clinical stages and criteria summarized in Figure 4 require hospital treatment^34^.

**FIGURE 5:**
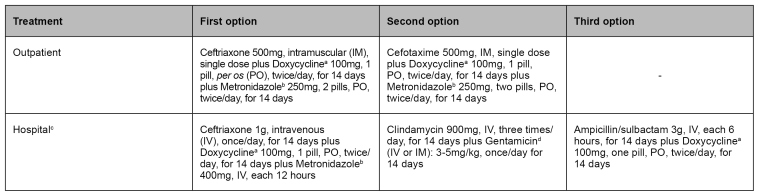
Pelvic inflammatory disease treatment.

**Source:** adapted from Clinical Protocol and Treatment Guidelines for Comprehensive Care for People with Sexually Transmitted Infections,2020^34^.

**Notes:** a) Doxycycline is a contraindication during pregnancy; b) According to professional evaluation, metronidazole may be stopped or not prescribed in light and moderate cases due to intolerance to use and the less important role of antibiotic therapy for anaerobic organisms in non-severe cases. Give information on not using alcoholic beverages during and after 24 hours of using metronidazole to avoid the disulfiram-like (Antabuse) effect; c) Parenteral use may be suspended 24 hours after the end of symptoms, and antimicrobial therapy must be extended to 14 days through the oral route; d) Gentamicin may be divided 2 to 3 times/day or reduced dose when clinically recommended.

Therapeutic schemes must present antimicrobial coverage for pelvic inflammatory disease etiological agents^34^ as shown in Figure 5. The antibiotic therapy must have a broad scope, be efficient against *N. gonorrhoeae, C. trachomatis,* and anaerobic organisms, especially *Bacteroides fragilis*, even if they are not confirmed in laboratory examination, and include bacterial vaginosis, Gram-negative bacteria, facultative bacteria, and streptococci^55^ ^,^ ^56^.

Among the broad-spectrum antibiotics with activity directed for such agents, beta-lactams, aminoglycosides, tetracyclines, lincosamides, and macrolides have been widely studied. They must be used in association with each other and can be administered through parenteral or oral route^42^ ^,^ ^43^. Parenteral use may be suspended 24 hours after the end of the symptoms, and antimicrobial therapy through oral or intramuscular route must be extended up to 14 days.

In most studies, doxycycline is the antibiotic of choice for treating *C. trachomatis*. The combination of clindamycin and gentamicin presents moderate activity against *N. gonorrhoeae* and *C. trachomatis in vitro,* and second-generation cephalosporins (cefoxitin or cefotetan) associated with doxycycline shows excellent *in vitro* results. The ampicillin/sulbactam association is also a good choice^55^ ^,^ ^56^. Due to a high level of bacterial resistance, quinolones are not used for treating *N. gonorrhoeae* in Brazil^57^.

For pelvic inflammatory disease treatment, PCDT indicates 500mg of ceftriaxone dosage in the recommended therapeutic schemes^34^. This dosage was chosen in a decision by Conitec, based on a cost-benefit and financial impact evaluation of using ceftriaxone 250mg in the national territory^58^. International protocols recommend ceftriaxone dosage as 250mg, depending on the availability of the medication and *N. gonorrhoeae* antimicrobial susceptibility local evidences^42^.

Clinical improvement is likely to occur within up to three days from the start of the antimicrobial treatment. The cure is based on the end of signs and symptoms and normalization of inflammatory markers in laboratory tests. A study showed that if the evaluation is carried out with bacteriologic criteria after thirty days, there is still one or more bacterial agents in 40% of women^7^. If the clinical picture persists or worsens, a diagnostic revision must be considered. Laparoscopy, resonance, or tomography examinations can be conducted in such cases^42^ ^,^ ^51^. Laparotomy is recommended in cases of adnexal masses unresponsive to treatment or rupturing. Culdotomy may be recommended if the abscess covers the bottom of the pouch of Douglas. In particular instances, tubo-ovarian abscesses can be drained with puncture guided through ultrasound^34^.

A return medical appointment to the outpatient follow-up must be scheduled in the first week, suggesting a second appointment 30 days after hospital discharge. In reproductive planning, the use or removal of hormonal contraceptives and IUD should be evaluated^34^ ^,^ ^59^. We recommend sexual abstinence for 30 days and condoms usage during intercourse after this time period. IUD removal must not be necessarily conducted in light and moderate cases of pelvic inflammatory disease^41^ ^-^ ^43^. Still, it must be considered if the user wishes to do it or if there is no clinical improvement after 72 hours from the antibiotic therapy or in cases of severe pelvic inflammatory disease^38^. However, when indicated, removal must only take place after two doses of the therapeutic scheme^60^. In such cases, using barrier or hormonal contraceptive methods must be recommended. 

The acute episode of pelvic inflammatory disease and its sequela results in high financial costs for women and the health system. Such expenses have increased substantially, considering many women will need assisted reproductive techniques^61^.

## SURVEILLANCE, PREVENTION, AND CONTROL

Like other STIs, pelvic inflammatory disease prevention and control must include counseling focused on the person and their sexual partners. The aim is self-recognition and minimizing risk factors for STI and new pelvic inflammatory disease episodes^62^. HIV, syphilis, and hepatitis B and C testing are recommended. In specific cases, vaccines for hepatitis A, hepatitis B, and HPV must be offered^34^.

All sexual partners must be counseled and evaluated, offering testing and vaccination. Sexual partners of up to two months before the diagnosis must be empirically treated for *N. gonorrhoeae* and *C. trachomatis* with intramuscular ceftriaxone 500mg and azithromycin 1g through oral route in a single dose^34^.

Pelvic inflammatory disease is not present in the Ministry of Health compulsory notification diseases, nor are the infections by *N. gonorrhoeae, C. trachomatis,* and *Mycoplasma genitalium* reported^63^. Despite this, the notifications can be carried out by the Federal District, states, and municipalities, depending on local decisions.

Pelvic inflammatory disease is a substantial public health problem. Mass screening for *N. gonorrhoeae* and *C. trachomatis* showed a reduction of pelvic inflammatory disease in women. The Center for Disease Control and Prevention of the United States recommends annual screening, primarily for sexually active women younger than 25 years and women older than 25 years with *C. trachomatis* infection risk, including their respective sexual partners. They also consider the possibility of screening men in scenarios with high prevalence and resources^42^ ^,^ ^64^. In the Brazilian setting, screening *N. gonorrhoeae* and *C. trachomatis* is recommended in some situations: first prenatal care medical appointments of pregnant women aged 30 or less, people with STI diagnosis, people living with HIV, sexual violence situations, people using HIV pre-exposure (PrEP) and post-exposure (PEP) prophylaxis and people with receptive anal sexual practice without condom use^34^.

A possibility to be assessed in future protocols is the inclusion of *M. genitalium* diagnosis for women with pelvic inflammatory disease, in addition to *N. gonorrhoeae* and *C. trachomatis* ^65^. European and North-American clinical guidelines included such investigation in women with pelvic inflammatory disease and men with non-gonococcal urethritides. Different studies showed *M. genitalium* association with cervicitis and pelvic inflammatory disease; however, there is no evidence of benefits for universal screening^41^ ^-^ ^43^.

## SPECIAL POPULATIONS

Pregnant women with pelvic inflammatory disease have a high risk of miscarriage, chorioamnionitis, and premature delivery^42^, and they must be hospitalized and undergo intravenous broad-spectrum antibiotic treatment immediately. Doxycycline and quinolones are a contraindication during pregnancy^34^.

Despite having a higher risk of pelvic inflammatory disease and complications, children, prepubertal adolescents, and women living with HIV have a similar clinical presentation and must be conducted in the same way as the general population^59^ ^,^ ^66^ ^,^ ^67^.
